# Epidemic modeling for misinformation spread in digital networks through a social intelligence approach

**DOI:** 10.1038/s41598-024-69657-0

**Published:** 2024-08-17

**Authors:** Sreeraag Govindankutty, Shynu Padinjappurath Gopalan

**Affiliations:** School of Computer Science Engineering and Information Systems, Vellore Institute of Technology, Vellore, 632014 India

**Keywords:** Epidemic model, Misinformation, Online digital networks, Sentiment, Social networks, Mathematics and computing, Applied mathematics, Computer science, Information technology

## Abstract

Online digital networks, including social networks, have significantly impacted individuals’ personal and professional lives. Aside from exchanging news and topics of interest, digital networks play an essential role in the diffusion of information, which frequently significantly impacts worldwide societies. In this paper, we present a new mathematical epidemic model for digital networks that considers the sentiment of solitary misinformation in the networks and characteristics of human intelligence that play an important role in judging and spreading misinformation inside the networks. Our mathematical analysis has proved the existence and validity of the system in a real-time environment. Considering the real-world data, our simulation predicts how the misinformation could spread among different global communities and when an intervention mechanism should have to be carried out by the policyholders. Our simulation using the model proves that effective intervention mechanisms by isolating the fake news can effectively control the spread of misinformation among larger populations. The model can analyze the emotional and social intelligence of groups frequently subjected to disinformation and disseminating fake news.

## Introduction

In the digital age, social media platforms have become integral to information dissemination, shaping public opinion and influencing societal discourse. However, these platforms also serve as fertile ground for the rapid spread of misinformation, presenting significant challenges to social cohesion, democratic processes, and public health. The propagation of false or misleading information across social networks has emerged as a critical area of study, intersecting fields such as computer science, sociology, psychology, and communication studies.

The digital and technological revolution post the twentieth-century has significantly impacted the world, affecting all societies and communities. Digital networks comprising online social networks are now attached to the life of every individual in this world. From sharing news and information to providing valuable consumer reviews, digital networks have become an inevitable part of human society worldwide, even in the healthcare sector. After the COVID-19 pandemic, when nations imposed lockdown measures to prevent the spread of the pandemic, digital networks proved to be an effective medium to connect across the world and assist individuals in their professional lives. This has exponentially increased the use of social networks among all categories of people during the pandemic era.

Social Intelligence—which is the ability to understand and navigate social situations effectively, is crucial in establishing connections and partnerships among individuals and organizations. Its significance was shown during the COVID-19 pandemic, when individuals, friends, and families were physically separated from one another. However, spreading fake news and misinformation is a significant obstacle to building trust and credibility^1^. Unfortunately, some individuals use digital networks to disseminate false information for their benefit, damaging the reputation and trust of individuals and communities^2^. People with lower emotional and social intelligence levels are likelier to share fake news and misinformation without considering its validity. Moreover, exposure to fake news and misinformation can impact an individual’s social intelligence, raising doubts about the authenticity and accuracy of shared information.

Several studies have showcased the detrimental impact of misinformation and fake news on society, especially in the healthcare sector^3^. Fake news and misinformation can adversely impact individuals’ and organizations’ reputations^4^, and studies have showcased that it can even mislead a group of people against the government or even against specific treatment practices^5^. False and bogus news during the COVID-19 pandemic has created additional apprehension among society related to medicational practices^6^, and incidents of vaccine hesitancy^7^ prove the same in several parts of the world^8^. At the same juncture, misinformation through digital networks was found to have affected the mental health of individuals^9^ upon constant exposure^10^. People exposed to misinformation were found to struggle with decision-making and problem-solving skills^11^.

Though the terms rumors, misinformation, disinformation, and fake news sound similar, each term has notable differences. Rumors are stories passed down from person to person, frequently with no evidence to back them up. Misinformation is similar in that it refers to disseminating incorrect information not purposely created to harm^12^. Disinformation, conversely, refers to the intentional fabrication and broadcast of erroneous content by individuals with the clear objective of inflicting harm^13^. Fake news is a type of deception designed to appear as actual news. It is widely disseminated on social media and can be difficult to discern from genuine news.

Misinformation in social media is characterized by its viral nature, often spreading faster and farther than accurate information. Several factors drive this phenomenon:

1. Echo chambers and filter bubbles: The digital and social media algorithms generally expose users to content that reinforces their beliefs, creating echo chambers that spread misinformation among like-minded communities^14^.
2. Emotional appeal: False information often elicits strong emotional responses, increasing the likelihood of sharing and engagement^15^.
3. Confirmation bias: Users are more likely to accept and spread information that confirms their preexisting beliefs or biases^16^.
4. Network effects: Social networks’ interconnected nature allows for the rapid, exponential spread of information across diverse user groups^17^.
5. Low barrier to content creation and sharing: The ease of creating and disseminating content on social media platforms facilitates the quick spread of unverified information.
6. Lack of fact-checking: The speed of information flow often outpaces traditional fact-checking mechanisms.

The dynamics of misinformation propagation in social networks resemble epidemiological models, where ‘infection’ represents exposure to and sharing of false information. This analogy has led to various mathematical models attempting to capture and predict the spread of misinformation, drawing parallels with disease transmission models. Understanding these interactions is critical for designing successful misinformation prevention techniques. These measures include increasing digital literacy, upgrading platform algorithms to detect and prevent the dissemination of misleading information, and conducting targeted interventions to disrupt the cycle of misinformation transmission.

Even though several mathematical epidemic models have evolved, most still need to explain the human nature of selection and social influence. The current models also fail to account for the reality that people may be motivated to spread false information for various reasons. Moreover, they also fail to explain the possibility of the creation of fake news by the super spreaders, where some viral misinformation can be propagated for a very long time. The models also assume that people are rational actors who make judgments based on careful consideration of the information. Considering this, the following contributions were made to this work.

1. Created a mathematical epidemic model by considering the human nature of selection and social intelligence with a restrained state and considering the power law property.
2. Mathematically proved the existence of the model in the real world.
3. Analyzed the stiffness of fake news in different communities using the model with real-world data.

The paper is divided into seven sections. The related epidemic models often used to explore social networks are discussed in the next section. In “Properties of the model”, we define and discuss the proposed model mathematically. “Analysis of the model” discusses the model’s attributes, which demonstrate the validity and existence of solutions in the real world. The significance of the model is explained using R inside the simulated environment and real-world data. “Simulation results” explains the simulation results. “Discussion” describes the findings and observed parameters, and “Conclusion and future scope” examines the study’s future scope and conclusion.

## Related works

Many investigations exploring the causes and mechanisms of how quickly misinformation spreads on various digital network platforms have been recommended. It was shown that people with fewer connections are necessary for rumors to spread swiftly^18^. Several mechanisms for fake news classification evolved in the recent past by pre-processing data along with the Glove-based word embedding process and machine learning models^19,20^. These methods provide better results in classifying fake news from actual news. However, the intervention mechanisms to counter the spread of misinformation should be applied at the right time in the right environment. The speed of propagation of fake news depends on several factors in each community. Regardless of whether a person is a student or a professional, rumors on social media can negatively impact their reputation. However, it can be pretty challenging to rebuild a reputation once it has been damaged^21^. Though preventing fake news from originating is inevitable, several epidemic models have been put forward to study and analyze the spread of information. Epidemic models in social networks are mathematical models used to investigate the transmission of infectious illnesses in social networks. We can better understand how misinformation in digital networks spreads and how to treat them with the aid of these models.

The growing pervasiveness of misinformation in digital networks necessitates advancements in understanding its spread^22^. Recent studies explore the role of artificial intelligence (AI) and large language models (LLMs) in misinformation campaigns, highlighting the potential for AI-driven content to exacerbate the issue^23^. Additionally, research is ongoing to develop improved methods for detecting and mitigating disinformation, mainly focusing on the challenges posed by the evolving nature of LLMs^24^. These studies underscore the importance of continuous research and development in the fight against misinformation in the digital age. Numerous research works examine the psychological elements that impact the receptivity to false information and the efficacy of therapies^25^. Studies reveal that people’s evaluations and online information sharing can significantly influence their emotional reactions and cognitive biases^26^. Studies also look at how pre-exposure to deceptive strategies might be used as an inoculation approach to increase community and individual resistance to deceptive efforts^27^. These results underline how crucial it is to consider psychological and technological factors when creating ways to counter false information as it raises anxiety in individuals, especially during the time of a crisis.

**Figure 1:**
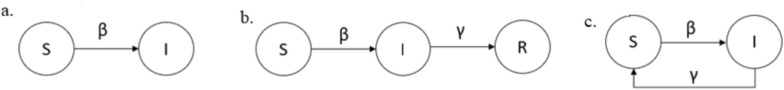
The traditional epidemic models (**a**) SI model. (**b**) SIR model. (**c**) SIS model.

Social network epidemic models are essential for misinformation as, unlike pathogenic viruses, misinformation spreads through social contacts and is susceptible to social biases and emotional reactions. Traditional epidemic models concentrate on infectious diseases. By taking into account these social and psychological factors and the network structure itself, these models enable researchers to investigate the spread of misinformation across networks. Network-based epidemic models, which study the propagation of the disease within the framework of the social network structure, have grown in favor more recently^28^. These models account for the various kinds of connections that people make with one another and how these connections impact the spread of the illness^29^. Diseases including HIV, Ebola, and COVID-19 have all been studied using network-based models. The SI, SIR, and SIS models are the most common^30^. These models divide the population of the entire network into susceptible (S), infected (I), and recovered (R) ones^31^. The Susceptible-Infected-Recovered (SIR) model, which has been expanded in many ways to include network topology, is one of the most extensively researched network epidemic models. The SIR model, for instance, can be altered to account for heterogeneous mixing patterns in the network^32^, including highly linked “hub” people who are more likely to spread the illness^33^. Figure 1 shows the traditional epidemic models where $\beta$ is the Infection rate from susceptible to the Infected state for all models, and $\gamma$ is the transmission rate from the Infected to the Recovered state and Infected susceptible state for the SIR and the SIS models respectively. The Susceptible-Infected-Susceptible (SIS) model holds that people can contract an infection more than once, and the Susceptible-Exposed-Infected-Recovered (SEIR) model, which includes an “exposed” state for people who have contracted an infection but are not yet contagious, are other examples of network epidemic models^34^. To explore the transmission of infectious diseases via social networks, the SEIR model has also been expanded to incorporate network structure^35^. In network SEIR models, people are linked not just by their disease status but also by their social ties^36^. Although individuals may have varying levels of connectedness and exposure to sick others^37^, the network structure can impact the dynamics of the disease’s transmission^38^.

The SEPNS model extends the SEIR model by splitting the Infected state into Positively Infected and Negatively Infected-based on the sentiment of rumors^39^. The Exposed node determines how likely a person is to believe misinformation and spread the same. People can move from the Susceptible state to the Exposed state by being exposed to misinformation. The Infected state was divided as Positively Infected and Negatively Infected, based on the sentiment of misinformation being propagated. However, the model failed to explain the neutral sentiment of misinformation and the possibility of a positively infected node becoming negatively infected and vice versa.

However, those who have lost interest in a topic should have been noticed by the model. It is less likely for someone who has lost interest in a subject to become susceptible to the same topic later on. Furthermore, the model also disregarded the human element of social influence and selection—vital in determining whether information is shared.

The SEDIS model is another development of the conventional SEIR epidemic model currently employed to research the transmission of infectious illnesses^40^. A new compartment designated Doubtful (D) is added to the SEDIS model to reflect people who have heard the story but have yet to be convinced that it is true. The Doubtful compartment is essential to spreading rumors since it determines whether they are true or false. The human tendency for selection—also known as the reality that people choose which information to trust and spread based on their preferences and biases—is another component of the SEDIS model. Based on the individual’s selection behavior, a function that captures the likelihood of a person moving from the Doubtful compartment to the Infected compartment has been put forth. The significance of considering the human nature of selection when analyzing the infodemics in social networks was emphasized. The SEDIS model can be termed as a pandemic model for social networks as it considers all the misinformation and fake news within the digital network as a single disease^41^. The model can be a helpful tool for comprehending and limiting the spread of false information in social networks since it offers a more realistic and accurate representation of the dynamics of rumor propagation. With $\alpha$ as the transmission rate from the Susceptible state to the Exposed state; $\beta _1$ and $\beta _2$ as the transmission rate from the Exposed state to the Doubter state and Infected state, respectively; $\gamma$ as the transmission rate from the Doubter state to the Infected state; and $\mu _1$, $\mu _2$, and $\mu _3$, as the transmission rate from the Exposed, Doubter and the Infected state to the Susceptible state; the model can be mathematically defined as follows;

1 $$\begin{aligned} \frac{ds}{dt}= & {} \mu _1e + \mu _2d + \mu _3i - \alpha s \end{aligned}$$

2 $$\begin{aligned} \frac{de}{dt}= & {} \alpha s - \beta _1e - \beta _2e - \mu _1e \end{aligned}$$

3 $$\begin{aligned} \frac{dd}{dt}= & {} \beta _1e - \gamma d - \mu _2d \end{aligned}$$

4 $$\begin{aligned} \frac{di}{dt}= & {} \gamma d + \beta _2e - \mu _3i \end{aligned}$$

However, whether the model could deal with a single misinformation or fake news within a community is still being determined. Though digital networks are engulfed with multiple misinformation and fake news, it is essential to understand the impact and spread of a single trending misinformation since a single bogus content can create unfortunate incidents within societies if no intervention mechanisms are carried out^42,43^.

## The SEDPNR model

By giving priority to the sentiment of rumors, we propose the SEDPNR (Susceptible-Exposed-Doubtful-Positively Infected-Negatively Infected-Restrained) model by splitting the conventional infected node based on the rumor sentiment along with adding a Restrained state into the SEDIS model, taking into account the necessity of an “end state” for disease transmission while considering a single trending misinformation or fake news in online digital networks. The Infected node is classified as Positively Infected and Negatively Infected based on the user’s attitude (whether the false information is viewed positively or negatively). As rumors and fake news typically spread only through solid emotions^44^, the infected node is divided according to these sentiments. The Restrained condition refers to people who have lost interest in the knowledge over time. Those who are no longer spreaders gradually fall into the Restrained state.

**Figure 2:**
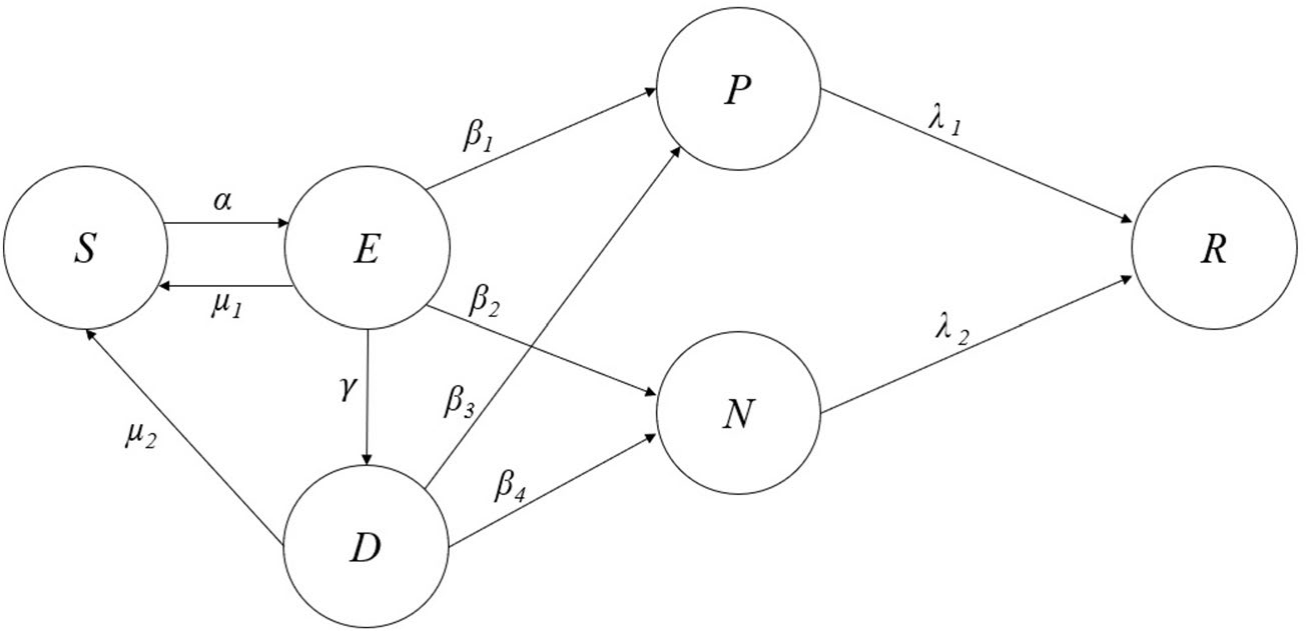
The proposed SEDPNR model.

Figure 2 depicts the SEDPNR model’s discrete compartmental diagram.

By observing the Fig. 2, *S*(*t*) denotes the individuals susceptible to the misinformation at time *t*, and *E*(*t*) refers to exposed individuals who met the trending fake news/misinformation. A person exposed to the trending misinformation can become Infected or Doubter *D*(*t*) depending upon his social and emotional intelligence at probability $\beta _1$ and $\beta _2$, respectively. The exposed individual can also return to the susceptible state if he lacks interest in the information. Individuals doubtful about the authenticity of information are said to be in the Doubter state. Such individuals can either become Infected after constant exposure to fake news or return to the Susceptible state upon losing interest or checking the authenticity of the information. *P*(*t*) refers to the positively infected individuals who spread misinformation in a positive tone at times *t*. Similarly, *N*(*t*) refers to the Negatively infected individuals who spread misinformation in negative sentiment. *R*(*t*) refers to the Restrained or muted individuals who lost interest in spreading the information. The loss of interest can happen either due to the course of time or by understanding that the information they shared is not genuine or fabricated. Regarding social network rumors and fake news, we ignore the word “Recovered” because it is essentially unattainable. An individual enters into the Restrained state only after becoming the spreader. This is because even though the Susceptible individual has returned from Doubter or Exposed state, he or she can become Infected (spreader) again if most of the community members share the misinformation and the topic attracts the community’s interest positively. The principle of social reinforcement and membership closure plays a crucial role in driving a community to share fake news^45^, even though the members within the community understand that the information they are about to share is bogus^46^.

The individuals in the Doubter state can also be named as distrustful members as they emphasize the individual’s general lack of trust in others, including the sources of information that spread rumors. A distrustful individual may be more likely to dismiss rumors as false, even if they have no concrete evidence to support this belief. The distrustful members in the network do not spread rumors among themselves, but they are susceptible to infection if they encounter similar rumors. They may return to the Susceptible state if they realize the information they received is untrustworthy. Considering the changing psychological trends, the Doubter condition is a new addition to traditional epidemic models. With newspapers, television channels, and fact-checking websites aggressively fighting against fake news, people are more likely to question the authenticity of the information. A person may choose to be in a doubter state and decide whether to accept or reject the rumor if the information loses clarity or authenticity. This aspect of human choice is unique to the Doubter condition. Considering $\alpha$ as the transition rate from the Susceptible to the Exposed state; $\beta _1$ and $\beta _2$ as the transition from the Exposed state to the Doubter state and Infected state, respectively; $\gamma$ as the transition rate from the Doubter state to the Infected State; $\lambda$ as the transition rate from the Infected state to the Restrained state; and $\mu _1$ and $\mu _2$ as the transition rate to the Susceptible state from the Exposed and Doubter state; the model can be mathematically explained as;

5 $$\begin{aligned} \frac{ds}{dt}= & {} \mu _1e + \mu _2d - \alpha s \end{aligned}$$

6 $$\begin{aligned} \frac{de}{dt}= & {} \alpha s - (\beta _1 + \beta _2 + \gamma + \mu _1 )e \end{aligned}$$

7 $$\begin{aligned} \frac{dd}{dt}= & {} \gamma e - (\beta _3 + \beta _4 + \mu _2 )d \end{aligned}$$

8 $$\begin{aligned} \frac{dp}{dt}= & {} \beta _1 e + \beta _3 d - \lambda _1 p \end{aligned}$$

9 $$\begin{aligned} \frac{dn}{dt}= & {} \beta _2 e + \beta _4 d - \lambda _2 n \end{aligned}$$

10 $$\begin{aligned} \frac{dr}{dt}= & {} \lambda _1 p + \lambda _2 n \end{aligned}$$

In more simple words, the transitions between states in our model closely mirror the behavior observed in real social networks:

1. S to E ($\alpha$): This represents users encountering misinformation for the first time, similar to seeing a shared post.
2. E to D ($\gamma$): Some users, after initial exposure, become skeptical and start questioning the information, much like when social media users fact-check or ask for sources.
3. E to P/N ($\beta _1$, $\beta _2$): This transition occurs when exposed users share the misinformation, either with a positive or negative sentiment, mirroring how people reshare posts with their commentary.
4. D to P/N ($\beta _3$, $\beta _5$): Doubters may eventually choose a side and start spreading the information, similar to how users might initially be skeptical but later convince themselves and share.
5. P/N to R ($\lambda _1$, $\lambda _2$): This represents users who stop spreading misinformation, perhaps due to fact-checking or platform interventions, analogous to users deleting posts or changing their stance.

For time *t*, the densities of all states can be represented as *S*(*t*), *E*(*t*), *D*(*t*), *P*(*t*) , *N*(*t*) and *R*(*t*). The specific evolution process for each state can be represented as;

11 $$\begin{aligned} \frac{ds}{dt} (t)= & {} (\mu _1e + \mu _2d - \alpha s)(t) \end{aligned}$$

12 $$\begin{aligned} \frac{de}{dt} (t)= & {} \alpha s (t) - (\beta _1 + \beta _2 + \gamma + \mu _1 )e (t) \end{aligned}$$

13 $$\begin{aligned} \frac{dd}{dt}= & {} \gamma e (t) - (\beta _3 + \beta _4 + \mu _2 )d (t) \end{aligned}$$

14 $$\begin{aligned} \frac{dp}{dt}= & {} (\beta _1 e + \beta _3 d - \lambda _1 p)(t) \end{aligned}$$

15 $$\begin{aligned} \frac{dn}{dt}= & {} (\beta _2 e + \beta _4 d - \lambda _2 n)(t) \end{aligned}$$

16 $$\begin{aligned} \frac{dr}{dt}= & {} (\lambda _1 p + \lambda _2 n)(t) \end{aligned}$$

According to network theory, a network with all its nodes being the same kind and possessing the same properties is called homogenous. The number of nodes with which each node has direct interaction is about the same, and this number follows the Poisson distribution. Under this assumption, the homogeneous network discusses the condition that *S*(*t*), *E*(*t*), *D*(*t*), *P*(*t*), *N*(*t*) and *R*(*t*) signify the population densities at time t that are susceptible, exposed, doubters, and infected, respectively. They fulfill the requirement:

17 $$\begin{aligned} S(t)+E(t)+D(t)+P(t)+N(t)+R(t)=N_t(t) \end{aligned}$$

where $N_t$ is the total population within the network community at time *t*. Based on the equations to 5 to 10; the algorithm for the model can be written as;

**Algorithm 1:**
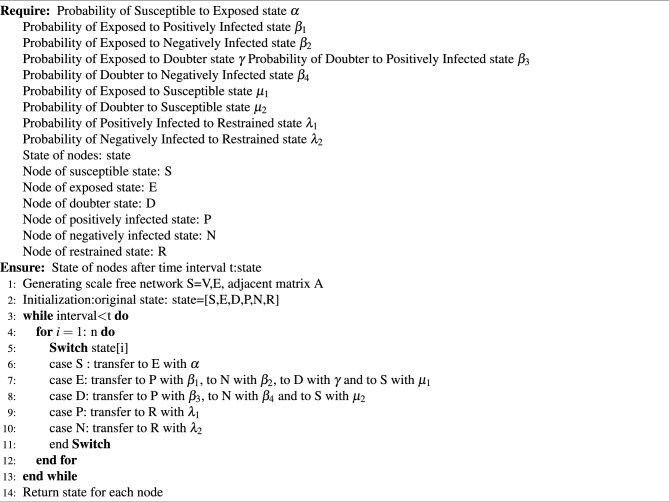
SEDPNR model

The time complexity of the SEDPNR Model algorithm is O(t $\times$ n), where t is the number of time intervals and n is the number of nodes in the network. This complexity arises from the nested loop structure, where the outer loop iterates t times, and for each iteration, we process all n nodes in the network. The first stage in constructing a scale-free network and initializing states has a complexity of O(N + E), where N is the number of nodes and E is the number of edges. However, the main loop structure precedes this stage in most real-world settings. It’s important to note that the algorithm’s time complexity is predictably linear for both the number of time intervals and the number of nodes, ensuring a consistent performance. The actual runtime, however, can be significant for large networks or extended time periods. The space complexity is O(n), as we need to store the state for each node.

We can say that this model captures several key phenomena observed in social media:

1. The rapid spread of misinformation through exposure and sharing (S to E to P/N).
2. The role of doubt and skepticism in potentially slowing spread (E to D).
3. The impact of emotional valence on sharing behavior (separate P and N states).
4. The potential for users to eventually stop spreading misinformation (P/N to R).

These dynamics align with observed behaviors on platforms like Twitter and Facebook, where misinformation can quickly go viral, face pushback from skeptical users, and eventually be contained through user awareness and platform interventions. The breakdown of assumptions underpinning the SEDPNR model is as follows;

1. Limited Attention and Processing: When people come across false information, they may fail to critically assess it or pay careful attention to it, which puts them in the “Exposed” condition.
2. Cognitive Biases: The Doubter category illustrates how people may believe or mistrust the message due to preexisting biases or incomplete knowledge.
3. Dynamic Belief States: The model acknowledges the potential of fact-checking or shifting opinions and permits people to go from accepting false information based on the sentiment or rejecting it.
4. Behavioral Change: The “Restrained” category suggests awareness-raising initiatives or interventions that reduce people’s propensity to disseminate false information.

## Properties of the model

This section investigates the SEDPNR model’s mathematical features, investigating its existence, stability, and behavior under various scenarios. We will first test the model’s ability to describe the dynamics of rumor propagation by establishing the presence of solutions for the system. The fundamental reproduction number, which assesses the potential for rumor spread, will be introduced next. Furthermore, we will determine the model’s positivity and validity, ensuring that the proportions of individuals remain within appropriate ranges. In addition, we will explore the model’s stability and the parameters under which the rumor dies out or endures indefinitely. This investigation will provide insights into the rumor’s long-term behavior and potential impact on the population.

### Existence of solution for the system

A solution to a system of equations is critical in many domains, including mathematics, physics, and engineering. A system can have no or several solutions, and knowing whether a solution exists has several advantages. It helps in aiding system validation, as an inconsistent system with no solution might lead to inaccurate conclusions or predictions. Additionally, it aids in the avoidance of errors that may occur when an inconsistent system is used in computations or simulations. Determining the presence of a solution is essential for verifying the precision and validity of mathematical, scientific, and engineering problems.

The existence of solution for the system is determined by using the Jacobian matrix. The Jacobian matrix is one mathematical tool used in calculus, especially vector calculus. All of the partial derivatives of a vector-valued function about its input variables are captured by what is effectively a square matrix. The square Jacobian matrix, *J*(*f*), has dimensions of *nxn*, where *n* is the number of input variables. Concerning each input variable $(x_1, x_2,\ldots , x_n)$, each element of the Jacobian reflects a partial derivative of one of the function’s output values $(f_1, f_2,\ldots , f_n)$.

#### Theorem 1

*For the system* (*S*(*t*), *E*(*t*), *D*(*t*), *P*(*t*), *N*(*t*), *R*(*t*)); *there always exists a solution at time* *t*.

#### Proof

The densities of each node can be denoted as *S*(*t*), *E*(*t*), *D*(*t*), *P*(*t*), *N*(*t*), *R*(*t*) at time *t*. The space of the system is $\Omega =(\{S(t),E(t),D(t),P(t),N(t),R(t)): S(t)\>0, E(t)\>0 , D(t)\>0, P(t)\>0, N(t)\>0 R(t)>0\}$. Let $U=\{S(t),E(t),D(t),P(t),N(t),R(t)): S(t)\>0, E(t)\>0 , D(t)\>0, P(t)\>0, N(t), R(t)>0\}$. Then we can say that $U \subseteq \Omega$ is an open set. The system can be expressed as $\frac{dx}{dt} = f$ where;

18 $$\begin{aligned} x= & {} (S(t),E(t),D(t),P(t), N(t), R(t)) \end{aligned}$$

19 $$\begin{aligned} f= & {} (f_1,f_2,f_3,f_4,f_5,f_6)^t \end{aligned}$$

The Jacobian matrix of the system is obtained as $J = \frac{\partial (u,v,w,x,y,z)}{\partial (s,e,d,p,n,r)}$ where;

20 $$\begin{aligned} u= & {} \mu _1e + \mu _2d - \alpha s \end{aligned}$$

21 $$\begin{aligned} v= & {} \alpha s - (\beta _1 + \beta _2 + \gamma + \mu _1 )e \end{aligned}$$

22 $$\begin{aligned} w= & {} \gamma e - (\beta _3 + \beta _4 + \mu _2 )d \end{aligned}$$

23 $$\begin{aligned} x= & {} \beta _1 e + \beta _3 d - \lambda _1 p \end{aligned}$$

24 $$\begin{aligned} y= & {} \beta _2 e + \beta _4 d - \lambda _2 n \end{aligned}$$

25 $$\begin{aligned} z= & {} \lambda _1 p + \lambda _2 n \end{aligned}$$

Upon solving, we get;

26 $$\begin{aligned} J = \begin{bmatrix} -\alpha &{} \mu _1 &{} \mu _2 &{} 0 &{} 0 &{} 0 \\ \alpha &{} -(\beta _1 + \beta _2 + \gamma + \mu _1) &{} 0 &{} 0 &{} 0 &{} 0 \\ 0 &{} \gamma &{} -(\beta _3 + \beta _4 + \mu _2) &{} 0 &{} 0 &{} 0\\ 0 &{} \beta _1 &{} \beta _3 &{} -\lambda _1 &{} 0 &{} 0 \\ 0 &{} \beta _2 &{} \beta _4 &{} 0 &{} \lambda _2 &{} 0 \\ 0 &{} 0 &{} 0 &{} \lambda _1 &{} \lambda _2 &{} 0 \end{bmatrix} \end{aligned}$$

We can deduce that the elements of the derived Jacobian matrix are, in nature, continuous. Hence $f:U \rightarrow R^6$ is a continuously differentiable map that explains the existence of a solution for the system. $\square$

### Basic reproduction number

In epidemic modeling, the basic reproduction number ($R_0$) is a significant metric that reflects the average number of infections induced by a single infectious individual in a susceptible network. In other words, it estimates a disease’s potential spread within a population. In simple words, the average number of people infected from infection from a single infected person in a community where everyone is susceptible is referred to as the basic reproduction number^47^. The user’s behavior can influence the value of $R_0$ in social networks, the network’s structure, and the features of rumors.

$R_0$ is essential in determining an outbreak’s severity and the interventions’ efficacy. If $R_0$ is less than one, it indicates that each infectious person is likely to infect fewer than one person on average, implying that the outbreak will eventually die out. If $R_0$ is more significant than one, the disease is more likely to spread quickly across the population and could produce an epidemic. To find the basic reproduction number ($R_0$) for the given model, we first identify the transmission and recovery terms in the system of equations. Then, we construct the next-generation matrix $F$ and the diagonal matrix of recovery rates $V$. Transmission terms:

$$\begin{aligned} \frac{ds}{dt}:&\quad \mu _1 e \\ \frac{de}{dt}:&\quad \alpha s \\ \frac{dd}{dt}:&\quad \gamma e \\ \frac{dp}{dt}:&\quad \beta _1 e \\ \frac{dn}{dt}:&\quad \beta _2 e \end{aligned}$$

Recovery terms:

$$\begin{aligned} \frac{dr}{dt}: \quad \lambda _1 p + \lambda _2 n \end{aligned}$$

Next-generation matrix $F$ and the diagonal matrix of recovery rates $V$:

$$\begin{aligned} F = \begin{bmatrix} 0 &{} 0 &{} 0 &{} \beta _1 &{} \beta _2 \\ 0 &{} 0 &{} 0 &{} 0 &{} 0 \\ 0 &{} 0 &{} 0 &{} 0 &{} 0 \\ 0 &{} 0 &{} 0 &{} 0 &{} 0 \\ 0 &{} 0 &{} 0 &{} 0 &{} 0 \end{bmatrix}, \quad V = \text {diag}(\lambda _1, \lambda _2, 0, 0, 0) \end{aligned}$$

V is a diagonal matrix with the values $\lambda _1$ and $\lambda _2$ on the main diagonal and zeros everywhere else. The basic reproduction number $R_0$ is the spectral radius of $FV^{-1}$. After calculations, we find:

27 $$\begin{aligned} R_0 = \max \left( \frac{\beta _1}{\lambda _1}, \frac{\beta _2}{\lambda _2}\right) \end{aligned}$$

So, the basic reproduction number $R_0$ for this social network epidemiological model is the maximum of the ratios of the transmission rates to the recovery rates for the positively and negatively infected compartments.

### Positivity and validity of the model

The positivity of a solution or system is significant in many domains. Being positive means that all components of the solution or system are greater than or equal to zero, which is significant for ensuring the validity of the proposed model and compatibility with natural laws. Since this model monitors the population for various classes, it is essential to show that all system parameters are non-negative.

#### Theorem 2

*Let* $\Omega = {(s,e,d,p,n,r) \in R^6 : s(0)>0,e(0)>0,d(0)>0,p(0)>0,n(0)>0 and r(0)>0}$ *then we can say that the solution* *s*(*t*), *e*(*t*), *d*(*t*), *p*(*t*), *n*(*t*), *r*(*t*) *of the model is positive for all* $t > 0$.

#### Proof

Taking the first compartment of the model from (5)

$$\begin{aligned} \frac{ds}{dt} = \mu _1e + \mu _2d - \alpha s \end{aligned}$$

which can be solved as

$$\begin{aligned} \frac{ds}{dt} \ge -\alpha s= & {} \frac{ds}{s} \ge (-\alpha )dt\\= & {} \int \frac{ds}{s} \ge \int (-\alpha )dt\\= & {} s(t) \ge s(0) e^{-\alpha t} \ge 0 \end{aligned}$$

Taking the second compartment of the model from (6);

$$\begin{aligned} \frac{de}{dt}= & {} \alpha s - \beta _1e - \beta _2e - \gamma e - \mu _1e\\= & {} \frac{de}{e} \ge -(\beta _1 + \beta _2 + \gamma +\mu _1)dt\\= & {} \int \frac{de}{e} \ge \int -(\beta _1 + \beta _2 + \gamma + \mu _1)dt\\= & {} e(t) \ge e(0).e^{-(\beta _1 + \beta _2 + \gamma + \mu _1)t} \ge 0 \end{aligned}$$

Similarly , taking third compartment from (7);

$$\begin{aligned} \frac{dd}{dt} = \gamma e - \beta _3 d - \beta _4 d - \mu _2 d \end{aligned}$$

On solving, we get;

$$\begin{aligned} d(t) \ge d(0)e^{-(\beta _3 + \beta _4 \mu _2)t} \ge 0 \end{aligned}$$

Finally, on solving the fourth, fifth, and sixth parts of the model from Eqs. (8) to (10); we get,

$$\begin{aligned} p(t)\ge & {} i(0)e^{-\lambda _1 t} \ge 0\\ n(t)\ge & {} i(0)e^{-\lambda _2 t} \ge 0\\ r(t)\ge & {} r(0)e^0 \ge 0 \end{aligned}$$

Since all parts of the model provide non-negative solution, we can say that the solution of the system is positive for all $t>0$ $\square$

.

#### Theorem 3

*Let* ${(S(0), E(0), D(0), P(0), N(0), R(0))>0}$. *The solution* *S*(*t*), *E*(*t*), *D*(*t*), *P*(*t*), *N*(*t*), *R*(*t*) *of the system is non-negative for* $t > 0$.

#### Proof

By contradiction let us assume that there exists $t_0 \in (0,t_1)$ such that any of the *S*(*t*), *E*(*t*), *D*(*t*), *P*(*t*), *N*(*t*), *R*(*t*) is negative. By the continuity of the solution there exists $t_0 \in (0,t_1)$ such that any of the ${S(t_0),E(t_0),D(t_0),P(t_0),N(t_0),R(t_0)}=0$. Without compromising generality, we suppose that $t_0$ is the shortest time with such a quality.

If $S(t_0)=0$ then ${E(t_0),D(t_0),P(t_0),N(t_0),R(t_0) \ge 0}$ is true implying that $\frac{ds}{dt}|_{t=0} = \mu _1 e(t_0 )+ \mu _2 d(t_0 )>0$ is true. By continuity of the problem, there exists a>0 such that S(t)is completely monotone growing in interval $(t_0 -a,t_0 +a)$.

Let $t_2 \in (t_0 -a,t_0)$. Then $I(t_2)<I(t_0)=0$ is true. Since $I(0)>0$ there exists $t_3 \in (0,t_2)$ such that $I(t_3)=0$ according to Bolzano’s theorem^48^, which contradicts the assumption of $t_0$^49^. If $E(t_0 )=0$; then $\frac{de}{dt}|_{t=t_0}= 0$ is true. It can be analyzed that $E(t)=0$ for all $t \ge 0$ is the solution with the starting value $E(0)=0$. But, $E(0)>0$ and $E(t_0 )=0$ contradicts the uniqueness of the solution.

We can similarly demonstrate that $D(t_0)=0,P(t_0)=0$, $N(t_0)=0$ and $R(t_0)=0$ which leads to a contradiction. Hence, we can say that the solution of the system *S*(*t*), *E*(*t*), *D*(*t*), *P*(*t*), *N*(*t*), *R*(*t*) is non-negative for $t>0$. $\square$

Moreover, we can also prove the stability of the model from the obtained equation for $R_0$ as practically the transmission rates are always positive. A model can be unstable if the basic reproduction number $R_0$ becomes negative^50^.

### Stability of the model and conditions under which the rumor dies or persists indefinitely

We can linearize the system of differential equations around the steady state when the number of exposed persons is zero in order to examine the stability of the rumor propagation model. The following matrix can be used to represent the linearized system:

$$\begin{aligned} \begin{bmatrix} -\alpha &{} \mu _1 &{} \mu _2 \\ \alpha &{} -\beta _1 - \beta _2 - \gamma - \mu _1 &{} 0 \\ 0 &{} \gamma &{} -\beta _3 - \beta _4 - \mu _2 \end{bmatrix} \end{aligned}$$

An eigenvalue of a square matrix A is a unique scalar value that, when multiplied by a non-zero vector *x*, produces another vector that points in the same direction (or a scalar multiple of that direction), albeit possibly with a different magnitude. Eigenvalues of the square matrix *A* are denoted by the Greek letter lambda, $\lambda$. This may be stated mathematically as $Ax = \lambda x$. In other words, an eigenvalue is a vector that scales a non-zero vector without affecting its basic orientation. The eigenvalues of this matrix determine the system’s stability. If all eigenvalues have negative real components, the system is stable, and the rumor will ultimately die. However, if at least one eigenvalue has a positive real part, the rumor will persist endlessly, indicating that the system is unstable.

We can use the following equation to examine the matrix’s eigenvalues:

$$\begin{aligned} \det (\delta I - A) = 0 \end{aligned}$$

where *I* is the identity matrix, *A* denotes the matrix representing the linearized system, and *delta* is the eigenvalue. Solving this equation for $\delta$ will give us the eigenvalues of the matrix. For the system defined, the eigenvalues of the matrix can be represented as;

$$\begin{aligned} \delta _1= & {} \frac{-\alpha + \sqrt{\alpha ^2 + 4 \mu _1 (\beta _1 + \beta _2 + \gamma + \mu _1)}}{2}\\ \delta _2= & {} \frac{-\beta _3 - \beta _4 - \mu _2 + \sqrt{(\beta _3 + \beta _4 + \mu _2)^2 + 4 \gamma (\beta _1 + \beta _2 + \gamma + \mu _1)}}{2}\\ \delta _3= & {} \frac{-\beta _3 - \beta _4 - \mu _2 - \sqrt{(\beta _3 + \beta _4 + \mu _2)^2 + 4 \gamma (\beta _1 + \beta _2 + \gamma + \mu _1)}}{2} \end{aligned}$$

If and only if all three eigenvalues are negative, the rumor will ultimately die; which means that:

$$\begin{aligned}{} & {} \alpha - \sqrt{\alpha ^2 + 4 \mu _1 (\beta _1 + \beta _2 + \gamma + \mu _1)}> 0\\{} & {} \beta _3 + \beta _4 + \mu _2 - \sqrt{(\beta _3 + \beta _4 + \mu _2)^2 + 4 \gamma (\beta _1 + \beta _2 + \gamma + \mu _1)}> 0\\{} & {} \beta _3 + \beta _4 + \mu _2 + \sqrt{(\beta _3 + \beta _4 + \mu _2)^2 + 4 \gamma (\beta _1 + \beta _2 + \gamma + \mu _1)} > 0 \end{aligned}$$

These conditions stand in for the limits of the stable area in the proposed model’s parameter space.

We can ascertain the effect of lower contact rates on rumor transmission by modeling the rumor’s spread with various values of $\alpha$. The social distancing strategies that result in the fewest number of people being exposed overall or the quickest time for rumors to circulate will be the most successful.

### Impact of misinformation and distrust on rumor propagation

We can expand the model to include characteristics linked to information sharing and trust to examine the influence of disinformation and mistrust of public health authorities on rumor dissemination. For instance, we may change the transition probabilities to account for the impact of false information on rumor susceptibility or create a new state to represent people who do not trust public health authorities.

We may replicate the spread of the rumor under various circumstances and compare the outcomes once the model has been expanded to include misinformation and mistrust. This will enable us to ascertain how these variables impact the spread of rumors and pinpoint populations more or less vulnerable to false information.

An example of how to change the model to include mistrust of public health authority is explained as follows;

28 $$\begin{aligned} \frac{ds}{dt}= & {} \mu _1e + \mu _2d - \alpha s \end{aligned}$$

29 $$\begin{aligned} \frac{de}{dt}= & {} \alpha s - (\beta _1 + \beta _2 + \gamma + \mu _1)e \end{aligned}$$

30 $$\begin{aligned} \frac{dv}{dt}= & {} \nu s - \tau v \end{aligned}$$

31 $$\begin{aligned} \frac{dd}{dt}= & {} \gamma e - (\beta _3 + \beta _4 + \mu _2)d \end{aligned}$$

32 $$\begin{aligned} \frac{dp}{dt}= & {} \beta _1 (1-\tau )ve + \beta _3 d - \lambda _1 p \end{aligned}$$

33 $$\begin{aligned} \frac{dn}{dt}= & {} \beta _2 (1-\tau )ve + \beta _4 d - \lambda _2 n \end{aligned}$$

34 $$\begin{aligned} \frac{dr}{dt}= & {} \lambda _1 p + \lambda _2 n \end{aligned}$$

where $\tau$ is the rate at which people grow distrustful, and v is the total number of distrustful people. In the Eqs. (32) and (33), the factor $(1-\tau )$ denotes the reduced sensitivity of distrustful people to misinformation.

We can ascertain the effect of mistrust on rumor propagation by modeling the spread of the rumor with various values of $\tau$. This method can also be expanded to consider additional elements like the influence of social media influencers or the frequency of false information sources linked to trust and information sharing.

### Finding equilibrium points

Equilibrium points in mathematical models, particularly differential equation models, are system states where some variables stay constant across time. An equilibrium point is a specific range of values for the model’s variables at which their rates of change approach zero. It is sometimes referred to as a steady state or a critical point. Put another way, all of the system’s variables cease to change, and the system stays in a steady state at the equilibrium point. To determine the equilibrium points, we must first set all of the rates of change in the system of differential equations of (5)–(10) to zero and then solve the resulting system of equations:

35 $$\begin{aligned}{} & {} \mu _1e + \mu _2d - \alpha s = 0 \end{aligned}$$

36 $$\begin{aligned}{} & {} \alpha s - (\beta _1 + \beta _2 + \gamma + \mu _1)e = 0 \end{aligned}$$

37 $$\begin{aligned}{} & {} \gamma e - (\beta _3 + \beta _4 + \mu _2)d = 0 \end{aligned}$$

38 $$\begin{aligned}{} & {} \beta _1 e + \beta _3 d - \lambda _1 p = 0 \end{aligned}$$

39 $$\begin{aligned}{} & {} \beta _2 e + \beta _4 d - \lambda _2 n = 0 \end{aligned}$$

40 $$\begin{aligned}{} & {} \lambda _1 p + \lambda _2 n = 0 \end{aligned}$$

We can identify the model’s equilibrium points by solving this set of equations. From Eq. (36), we can solve for *e* as;

$$\begin{aligned} e = \frac{\alpha s}{\beta _1 + \beta _2 + \gamma + \mu _1} \end{aligned}$$

Substituting this expression for *e* into the Eqs. (35), (37), and (38), we get:

$$\begin{aligned}{} & {} \mu _1\left( \frac{\alpha s}{\beta _1 + \beta _2 + \gamma + \mu _1}\right) + \mu _2d - \alpha s = 0\\{} & {} \gamma \left( \frac{\alpha s}{\beta _1 + \beta _2 + \gamma + \mu _1}\right) - (\beta _3 + \beta _4 + \mu _2)d = 0\\{} & {} \beta _1\left( \frac{\alpha s}{\beta _1 + \beta _2 + \gamma + \mu _1}\right) + \beta _3 d - \lambda _1 p = 0 \end{aligned}$$

Now, from (40), we can solve for *p* as;

$$\begin{aligned} p = \frac{-\lambda _2 n}{\lambda _1} \end{aligned}$$

Substituting this expression for p into the Eq. (39), we get:

$$\begin{aligned}{} & {} \beta _2 e + \beta _4 d - \lambda _2 n = 0\\{} & {} \beta _2 e + \beta _4 d + \lambda _1 n = 0 \end{aligned}$$

Adding these equations together;

$$\begin{aligned} (\beta _2 + \beta _4)e + (\beta _4 + \mu _2)d = 0 \end{aligned}$$

Substituting the expression for *e* from the previous steps;

$$\begin{aligned} (\beta _2 + \beta _4)\left( \frac{\alpha s}{\beta _1 + \beta _2 + \gamma + \mu _1}\right) + (\beta _4 + \mu _2)d = 0 \end{aligned}$$

Solving for *d*, we get;

$$\begin{aligned} d = \frac{-(\beta _2 + \beta _4)(\alpha s)}{(\beta _4 + \mu _2)(\beta _1 + \beta _2 + \gamma + \mu _1)} \end{aligned}$$

Substituting this expression for *d* into the expression for *p* from previous steps, we get;

$$\begin{aligned} p = \frac{-\lambda _2 n}{\lambda _1} \end{aligned}$$

$$\begin{aligned} p = \frac{\lambda _2(\beta _2 + \beta _4)(\alpha s)}{(\beta _4 + \mu _2)(\beta _1 + \beta _2 + \gamma + \mu _1)\lambda _1} \end{aligned}$$

Substituting this expression for *p* into the Eq. (38), we get:

$$\begin{aligned}{} & {} \beta _1 e + \beta _3 d - \lambda _1 p = 0\\{} & {} \beta _1\left( \frac{\alpha s}{\beta _1 + \beta _2 + \gamma + \mu _1}\right) + \beta _3 d - \lambda _1 p = 0 \end{aligned}$$

Finally; upon solving for s, we get:

41 $$\begin{aligned} s = \frac{\mu _1 (\beta _3 + \beta _4 + \mu _2)(\lambda _1 + \lambda _2)}{\mu _1 \beta _3 + \mu _2 \lambda _1} \end{aligned}$$

Now; upon subsitituting the expressions for *e, d, p* and *n* from the previous steps into the original system of equations, we get:

42 $$\begin{aligned} e= & {} \frac{\alpha (\beta _3 + \beta _4 + \mu _2)(\lambda _1 + \lambda _2)}{\alpha \beta _3 + (\beta _1 + \beta _2 + \gamma + \mu _1)\lambda _1} \end{aligned}$$

43 $$\begin{aligned} d= & {} \frac{\gamma (\lambda _1 + \lambda _2)(\beta _1 + \beta _2 + \gamma + \mu _1)}{\gamma \beta _1 + (\beta _3 + \beta _4 + \mu _2)\lambda _2} \end{aligned}$$

44 $$\begin{aligned} p= & {} \frac{(\mu _1 \beta _3 + \mu _2 \lambda _1)(\lambda _2 p + \lambda _1 n)}{\beta _1 \lambda _2} \end{aligned}$$

45 $$\begin{aligned} n= & {} \frac{(\beta _1 + \beta _2 + \gamma + \mu _1)\lambda _2)(\lambda _1 p + \lambda _2 n)}{\beta _2 \lambda _1} \end{aligned}$$

which are the equilibrium points of the system for a clustered network. This equilibrium point represents a state where the rumor is persistently circulating in the population. The values of the variables in this equilibrium point will depend on the values of the model parameters.

We may determine the effect of network clustering on rumor prevalence by comparing the values of *p* in the two equilibrium points. If the value of *p* is greater in the clustered network’s equilibrium point, then clustering is thought to aid rumor spread. In contrast, if the value of *p* is smaller in the clustered network’s equilibrium point, clustering is thought to impede rumor spread.

- If $\beta _1 > \beta _4,\beta_2$, then *p* will be higher in the equilibrium point for the clustered network, indicating that clustering facilitates rumor propagation among positively believing individuals.
- If $\beta _1 < \beta _4, \beta_2$, then *p* will be lower in the equilibrium point for the clustered network, indicating that clustering hinders rumor propagation among positively believing individuals.
- Similarly, if $\beta _2 > \beta _3 , \beta_1$, then n will be higher in the equilibrium point for the clustered network, indicating that clustering facilitates rumor propagation among negatively believing individuals.
- If $\beta _2 < \beta _3, \beta_1$, then n will be lower in the equilibrium point for the clustered network, indicating that clustering hinders rumor propagation among negatively believing individuals.

This means that if the infection rate within a belief group is higher than the overall network rate, clustering can amplify the spread of misinformation within that specific group. This helps us understand social network structure’s potential influence on spreading misinformation based on the underlying dynamics of belief-specific infection rates. This is because the parameters $\beta _1$ and $\beta _2$ represent the rates at which exposed individuals convert positively and negatively believing individuals, respectively. Similarly, $\beta _3$ and $\beta _4$ represent how distrustful individuals convert to positively and negatively believing individuals.

A system is considered stable when it maintains its proximity to the equilibrium point across time, having started from a condition that is near it. The system won’t diverge greatly, even in response to minor perturbations. An equilibrium point is considered asymptotically stable if the system, from any nearby state, stays near and progressively moves closer to the equilibrium point over time. The system eventually approaches and stays arbitrarily near the equilibrium point.

#### Theorem 4

*The SEDPNR model is globally asymptotically stable.*

#### Proof

To establish the global asymptotic stability of the SEDPNR model, we introduce a Lyapunov functional and prove its monotonicity along the system’s trajectories. A Lyapunov functional is an effective tool for evaluating the stability of a dynamic system. A Lyapunov function is defined on the state space of a dynamical system and is a scalar function, which means it produces a single numerical value^51^. All different setups or states that the system may be in are represented by this state space. The designated Lyapunov functional for the SEDPNR model can be written as:

46 $$\begin{aligned} V(s, e, d, p, n, r) = s^2 + e^2 + d^2 + p^2 + n^2 + r^2 \end{aligned}$$

By examining the derivative of the Lyapunov functional concerning time and replacing the equations for the rates of change in our SEDPNR model, we can demonstrate that this functional decreases along the model’s trajectories.

47 $$\begin{aligned} \begin{aligned} V'(s, e, d, p, n, r)&= 2s(\mu _1e + \mu _2d - \alpha s) + 2e(\alpha s - (\beta _1 + \beta _2 + \gamma + \mu _1)e) \\&\quad + 2d(\gamma e - (\beta _3 + \beta _4 + \mu _2)d) + 2p(\beta _1e + \beta _3d - \lambda _1p) \\&\quad + 2n(\beta _2e + \beta _4d - \lambda _2n) + 2r(\lambda _1p + \lambda _2n) \\&= 0 \end{aligned} \end{aligned}$$

Since the derivative of the Lyapunov functional is zero, we can infer that the functional maintains a constant value along the trajectories of the SEDPNR model. This indicates that the SEDPNR model exhibits global asymptotic stability, signifying that the system will ultimately reach an equilibrium where the proportions of susceptible, exposed, doubtful, positively infected, negatively infected, and restrained individuals will remain unchanged. $\square$

## Ethical Considerations

The study of misinformation diffusion and the development of models like SEDPNR raise critical ethical considerations. Misinformation can have a significant negative impact on individuals and society as a whole. Therefore, it is crucial to ensure this model’s responsible development and application. Some of the significant ethical considerations are as follows;

- Privacy, transparency, and data protection: All data-gathering activities, including surveys and text-mining social networking sites, must follow strict privacy laws. Users’ anonymity and informed consent ought to be top priorities. Additionally, it is crucial to ensure transparency in its development and application. This includes documenting the model’s assumptions, limitations, and potential biases. Moreover, the model’s predictions should be interpretable, allowing users to understand the reasoning behind its outputs.
- Potential Misuse of the Model: The SEDPNR model can be a powerful tool for understanding misinformation spread. However, it is essential to consider how the model could be misused. For example, the model could identify and target specific groups with misinformation campaigns, potentially exacerbating the problem.
- Social Responsibility: Researchers have a responsibility to use their findings for good. The SEDPNR model should be utilized to develop strategies for mitigating misinformation spread, not for amplifying it. Collaboration with policymakers and social media platforms is essential to ensure the model’s ethical application.

By acknowledging and addressing these ethical considerations, the SEDPNR model can be used as a force for positive change in the fight against misinformation.

## Analysis of the model

### Analysis using R

**Figure 3:**
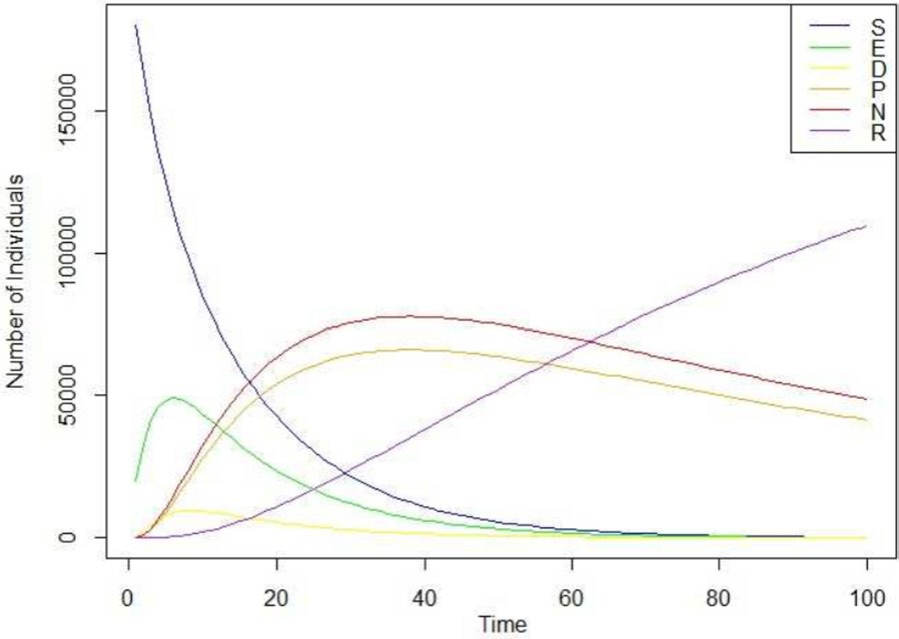
Simulation of SEDPNR Model with 200,000 individuals under random transmission rate for 100 days.

The computational analysis was done using R language, an open-source language enhanced by the developer community that contributes to its development. Based on Algorithm 1, the computational simulation was made with a sample population of 200,000 individuals under random probabilistic values, assuming that a negative tone of rumor spreads more than that of positive sentiment. Figure 3 shows the simulation for the SEDPNR model for 100 days based on the mathematical definition and algorithm 1. The figure shows that the Power law property can graphically be identified for the Susceptible and the Infected population, which showcases super-spreaders and idle populations within the network. A detailed comparison with other similar epidemic models is mentioned in the next section. A power law is a degree distribution in graphs where the probability of a node having k connections (i.e., its degree) is proportional to $k(-)$, where it is a constant known as the power law exponent. The power law describes how connections among members of social networks are distributed. This suggests that a tiny group of nodes, often of prominent and influential people, have many connections^52^. The remaining nodes, on the other hand, are proportionally less connected. The way connections are distributed inside a network might affect how rumors spread. Nodes with more connections can act as super-spreaders by quickly disseminating information to a large audience.

A power law distribution means that a few nodes in a network have a significant number of connections (or degrees). In contrast, the majority of nodes have a small number of connections. This pattern is typical in real-world networks like social networks, the internet, web pages, and biological networks^53^. Power law distributions in the spread of infectious diseases have been seen, and they have significant implications for modeling and understanding epidemics^54^. Studies also show that the distribution of $R_0$ values for many infectious diseases follows a power law distribution^55^, with a few responsible for a substantial share of secondary infections^56^. This pattern has significant implications for epidemic modeling and control since focusing on a limited number of highly infectious individuals, called “super-spreaders,” may effectively limit disease spread. Moreover, a power law distribution in $R_0$ values may help explain the observed variation in epidemic patterns, where some outbreaks are small and contained.

### Analysis with real-world data

Previous studies showcase stiffness analysis using the SIR model, which was conducted to anticipate the spread of false news in social networks in specific nations^57^. With $\phi$ as the recovery rate and $\omega$ as the infection rate, these parameters are related to two indices often used to assess global communities’ social, economic, and cultural performance. Generally,

48 $$\begin{aligned} \phi = \frac{i}{10} and \omega = \frac{h}{100} \end{aligned}$$

**Figure 4:**
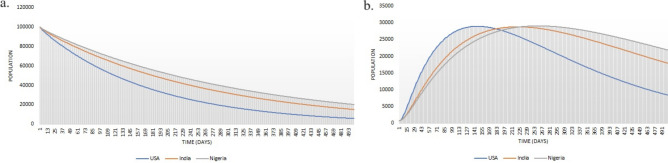
Susceptible (**a**) and infected population (**b**) with values of 2022 on 100,000 individuals.

Here *i* refers to the internet penetration index of the nation^58^ and *h* refers to the HDI of each nation^59^. Generally, $\phi$ is less than $\omega$ since spreading bogus content is easier than reinforcing the truth^60^. Table 1 demonstrates the 2022 values of $\phi$ and $\omega$ for the randomly selected high, medium, and low HDI nations, i.e., USA, India, and Nigeria. Figure 4 report each nation’s susceptible and infected population (the combination of positive and negatively infected nodes), assuming the community size in each nation as 100,000 individuals and with an additional assumption that 99.9% of the population is initially susceptible, with no restrained ones. The analysis period was kept for 500 days. By analyzing the misinformation trend in Fig. 4, we highly recommend the need for a sustainable and effective intervention mechanism within digital networks for compacting fake news and misinformation in nations with relatively high internet penetration rates along with lower HDI values.

**Table 1:** Values of constants $\phi$ and $\omega$ for USA, India and Nigeria, referring to 2022.

Country	*h*	*i*	$\phi$	$\omega$
USA	0.921	96%	0.009	0.096
India	0.633	58%	0.006	0.058
Nigeria	0.535	63%	0.005	0.063

According to observations, the susceptible population decreases quickly in nations with higher internet penetration values than those with low HDI and low internet penetration values. However, from Fig. 6, it is also evident that the infected population decreases rapidly for nations with higher HDI values. This highlights the necessity of intervention measures against misinformation in low HDI nations compared to high HDI nations, as spreaders tend to be active for a longer time in low HDI nations than in high HDI nations. Additionally, the susceptible and infected populations exhibit a power law during their downward slope, emphasizing the importance of super-spreaders within the community.

#### Case study: Kochi fire incident

**Figure 5:**
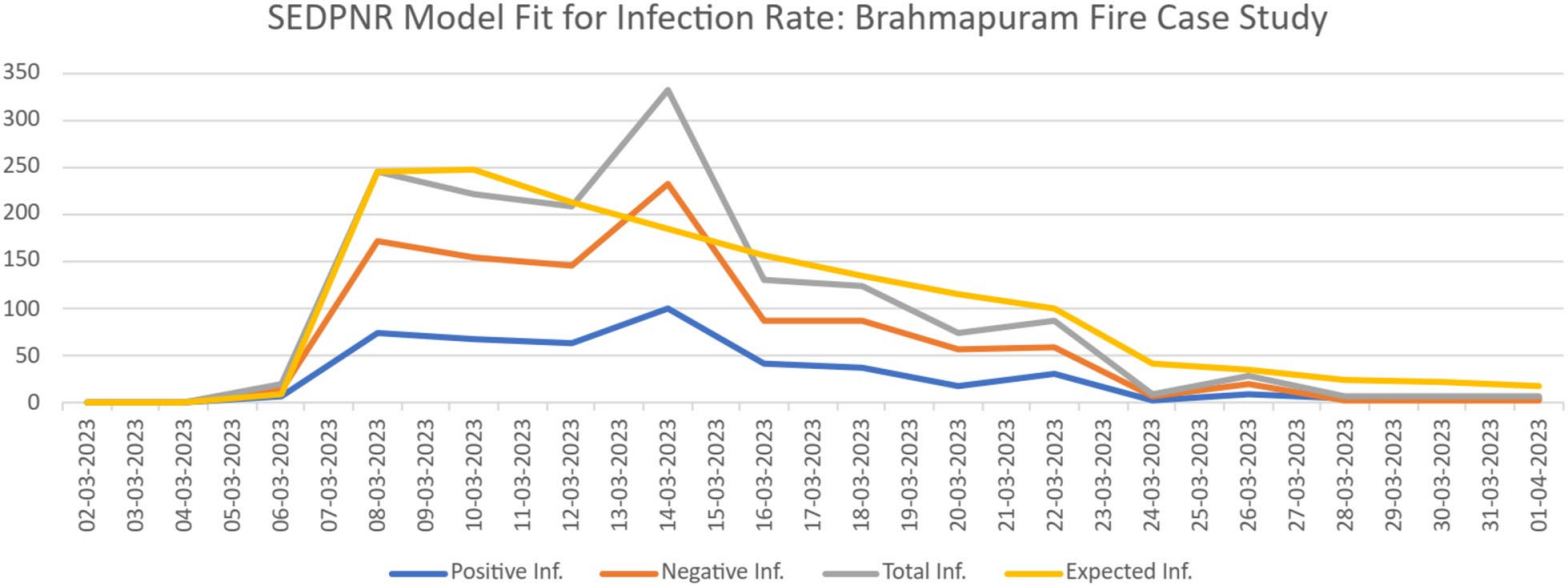
SEDPNR model fit for information spread during the 2023 Kochi—Brahmapuram fire incident.

Figure 5 presents the SEDPNR Model fit for the infection rate during the Brahmapuram fire incident in Kochi, Kerala, India^61,62^. The data was collected from all the tweets—based on hashtags and keywords about the incident from March 1 to April 2, 2023. The x-axis represents the number of daily tweets, while the y-axis shows the event’s timeline.

The graph illustrates four key metrics: positive infection rate (blue line), negative infection rate (orange line), total infection rate (grey line), and the expected infection rate as predicted by our SEDPNR model (yellow line). The data reveals a sudden surge in tweet activity starting around March 7–8, coinciding with the outbreak of the fire at the Brahmapuram waste treatment plant. This initial spike likely represents the rapid spread of information as news of the fire broke.

A notable peak in total infection rate is observed on March 14–15, corresponding to a secondary fire outbreak at the site. This exceptional case triggered a renewed surge in social media activity, underscoring the model’s sensitivity to real-world events. The graph shows that negative sentiment (orange line) generally outweighed positive sentiment (blue line) throughout the incident, possibly reflecting public concern and criticism over the fire’s environmental and health impacts.

Following the peak, we observe a gradual decline in infection rates across all categories, indicating a waning of public interest or potentially the containment of the fire situation. The expected infection rate (yellow line) fits reasonably well with the total infection rate (grey line), particularly in capturing the overall trend and significant fluctuations. However, some discrepancies are noted, especially around the March 14–15 peak, which may be attributed to the unexpected nature of the secondary fire outbreak.

This case study underscores the SEDPNR model’s robust capability to predict general trends in information spread during a localized environmental crisis. It also highlights areas for potential refinement to better account for sudden, unpredictable events within an ongoing incident, instilling confidence in its predictive power.

#### Sentiment analysis during crisis period

**Figure 6:**
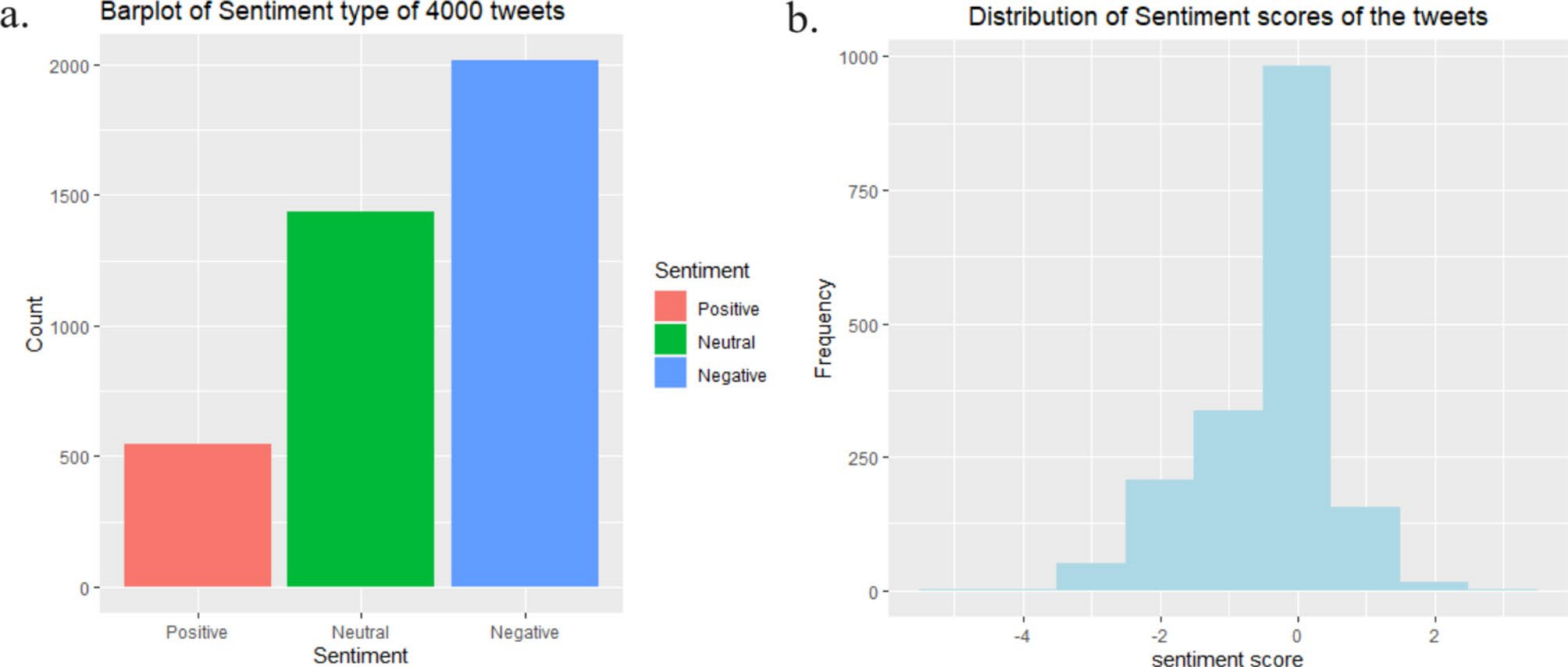
An examination of public sentiment toward the Taliban during their takeover of Afghanistan [(**a**) at the time of the takeover, (**b**) on Twitter following the Pakistani government’s pro-Taliban declaration].

Studies have shown a significant increase in negative sentiment in social networks during or after a crisis compared to before the crisis^63^. Our analysis conducted during the Taliban takeover in Afghanistan proves the same. Figure 6 shows the sentiment analysis after extracting 4000 random tweets with the hashtag *#taliban* on September 2021 while the Taliban gained control in Afghanistan. A significantly dominant negative sentiment was observed.

Figure 6 also shows the sentiment analysis results on how people reacted when the Pakistani government supported the Taliban, mentioning that they initiated a dialogue with the Taliban to encourage them to form an inclusive government^64^. Even though the news has a positive/neutral tone, people reacted negatively. This proves that negative sentiment can be dominated during a crisis or when a dramatic change happens to the lifestyle of a community.

## Simulation results

The positivity and boundedness of the epidemic model will be verified first in this section. Following that, two distinct control strategies are offered. One technique uses no intervention mechanisms, whereas the other uses intervention mechanisms.

### Positivity and boundness

A bounded model is one where the variables or solutions remain within a finite range. Sensitivity to parameters is crucial to understanding the model’s behavior and limitations. Here is a breakdown of how the model might be sensitive to various parameters: A higher $\alpha$ signifies a faster spread of misinformation. The model would be sensitive to this parameter, with a slight increase in $\alpha$ leading to a significant rise in “positive infected” individuals. Similarly, a higher value of infection rates signifies faster spread and vice versa. This implies that in a community where the data given aligns with the community’s interests, the misinformation is likely to spread more quickly.

**Figure 7:**
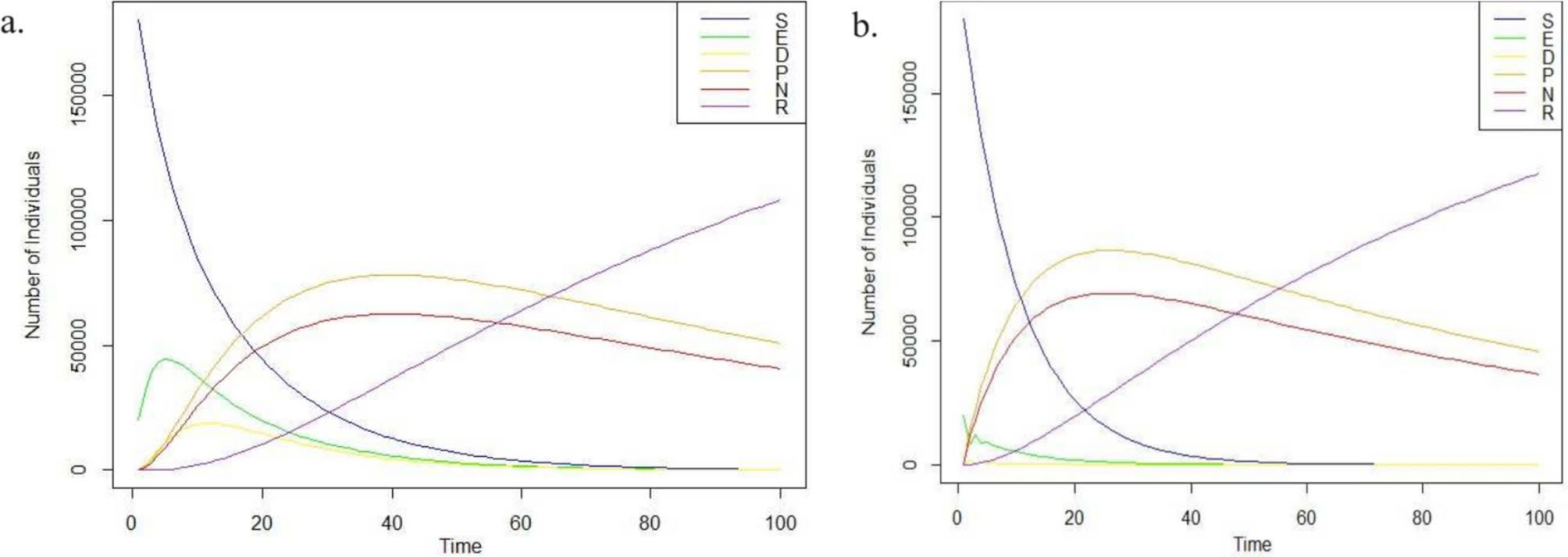
SEDPNR model—(**a**) At lower transmission rate. (**b**) At a higher transmission rate.

In this simulation, two cases are developed for the analysis. We began both simulations with a population of 200,000 (represented by *S*(0) = 199,990). A small number of individuals were initially exposed (*E*(0) = 10) and doubtful (D(0) = 10), with an equal number leaning towards positive (*P*(0) = 5) or negative (*N*(0) = 5) beliefs about the misinformation. Nobody was initially restrained (*R*(0) = 0). More precisely, we can say that the initial state of the system as $[S(0), E(0), D(0), P(0), N(0), R(0)]^T = [199,990, 10, 10, 5, 5, 0]^T$. In the first scenario, we assumed a relatively balanced flow of information. This means people were exposed to information that challenged misinformation at a similar rate to exposure from misinformation sources (represented by lower $\beta$ values: $\beta _1$ = 0.075,$\beta _2$ = 0.06, $\beta _3$ = 0.075, $\beta _4$ = 0.06). In the second case, the infection rate is re-set as $\beta _1$ = 0.75, $\beta _2$ = 0.6, $\beta _3$= 0.75 and $\beta _4$ = 0.6. The remaining system parameters and population remain constant. Figure 7 depicts the simulation outcomes at a lower and increased infection rate. The figures show that all subpopulations are bounded, and the total population is positive. In Fig. 7, we can also observe that the susceptible population quickly comes to a null value as the infection rate increases. Moreover, the relevance of the “Doubter” state goes down in a network where the infection rate is considerably high.

#### Theorem 5

*The SEDPNR model is bounded, and the total population is always positive for the model*.

#### Proof

Considering the Eqs. (5) to (10), the sum of the proportions is defined as;

$$\begin{aligned} S = s + e + d + p + n + r \end{aligned}$$

By utilizing mathematical techniques, we can derive an equation representing the rate at which the sum of the proportions changes.

$$\begin{aligned} \frac{dS}{dt} = \mu _1e + \mu _2d - \lambda _1p - \lambda _2n \end{aligned}$$

Since all the parameters are non-negative, we have

49 $$\begin{aligned} \mu _1e + \mu _2d - \lambda _1p - \lambda _2n \ge 0 \end{aligned}$$

This suggests that the sum of the proportions always experiences a positive change, indicating that it constantly increases. As the initial value of the sum falls between 0 and 1, it will continue to stay within this range. This guarantees that the individual proportions (*s*, *e*, *d*, *p*, *n*,  and *r*) must also be confined to 0 and 1. Ultimately, this is a significant assurance for the bounds of each proportion. $\square$

The SEDPNR model’s predictions are highly sensitive to parameters governing information flow. A slight increase in the rate at which people encounter misinformation ($\alpha$) can significantly increase the number of Infected individuals, potentially causing a more extensive and faster outbreak. This can be observed by setting the parameter values as $\alpha$=0.1, $\gamma$=0.025, $\beta _1$=0.05, $\beta _2$ = 0.05, $\beta _3$=0.05, $\beta _4$=0.05, $\lambda _1$=0.035, $\lambda _2$= 0.035, $\mu _1$ = 0.01, and $\mu _2$ = 0.1; and $[S(0), E(0), D(0), P(0), N(0), R(0)]^T = [299,990, 0, 0, 5, 5, 0]^T$. At this rate, the peak value of the total infection rate stands at 116,354 (at step 31). Similarly, on setting $\alpha$ to higher values, say 0.2, the peak infection rate stands at 141,874 at step 25, signifying an increase in infection rate

Similarly, the relative values of $\gamma$ (becoming a doubter) and $\beta _1$, $\beta _2$, $\beta _3$, and $\beta _4$ (becoming infected) are crucial. If $\gamma$ is close to the infection rates, a small shift can significantly impact the number of doubters vs the infected ones. A higher $\gamma$ relative to infection rates suggests a smaller outbreak as more people become susceptible.

### Evaluation of intervention strategies

It is an efficient technique to regulate misinformation and rumors by increasing fake news screening and cutting off the source of infection, according to the features of misinformation dissemination in social networks. We believe isolating fake news, like infectious disease, can generate fruitful results. The isolation is intended to keep fake news from exposure to other people and keep the content away from vulnerable groups. Once isolation measures are implemented, the system model parameters will be reduced.

Evaluating intervention strategies within the SEDPNR model is significant for several reasons. By replicating their impacts in the model, we can learn more about how various initiatives affect the dissemination of false information in the real world. This enables social media platforms and policymakers to create and execute more potent plans to counteract disinformation operations. Evaluating intervention strategies within the SEDPNR model allows us to move beyond simply understanding how misinformation spreads to actively exploring ways to mitigate its impact. It provides a valuable tool for developing and testing strategies that can make a real difference in the fight against misinformation.

**Figure 8:**
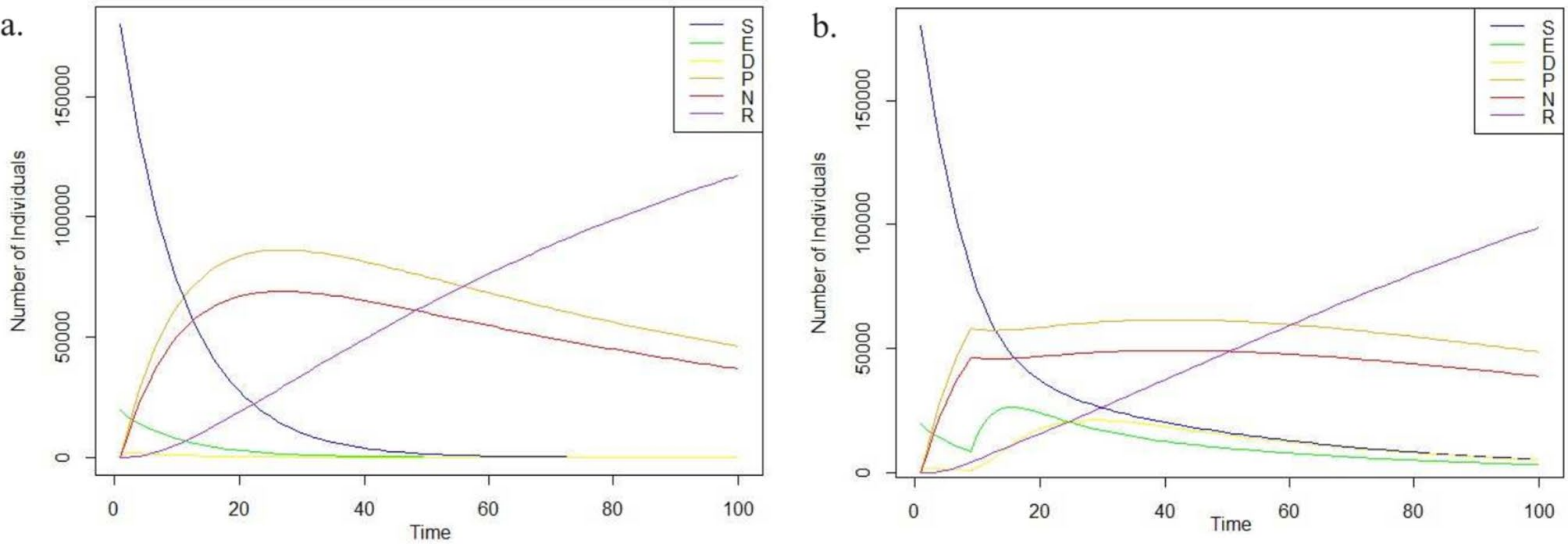
Population under the SEDPNR model without intervention.

Figure 8 shows the rumor propagation using the SEDPNR model with the values of parameters as $\alpha$ = 0.1, $\gamma$ = 0.1, $\beta _1$ = 0.5, $\beta _2$ = 0.4, $\beta _3$ = 0.5, $\beta _4$ = 0.6, $\lambda _1$ = 0.01, $\lambda _2$ = 0.01, $\mu _1$ = 0.05, and $\mu _2$ = 0.05 and the initial state is set as $[S(0), E(0), D(0), P(0), N(0), R(0)]^T$ = $[199,990, 10, 10, 5, 5, 0]^T$. In the first condition, we assume that no intervention mechanisms are taken.

In the second case, for the same population and transmission rate, we consider an intervention mechanism starting from day 10 for 30 days, which reduces the infection rate by 25%. From the figure, we can understand that misinformation spreads swiftly if no intervention measures are taken, and the number of affected people peaks quickly. However, once the relevant policyholders have taken the necessary intervention measures to reduce the spread by just 25%, the transmission spread is significantly reduced.

## Discussion

Online digital networks are computer networks that allow users to share digital information over the internet. These networks provide real-time communication, cooperation, and idea sharing, opening new avenues for socialization and innovation. They are, nevertheless, highly dynamic and vulnerable to internet abuse and misinformation. As a result, several online digital networks have established standards and guidelines to encourage responsible use, such as content control and user reporting^65^. Individuals, corporations, and society have benefited from new opportunities and problems from online digital networks^66^. The former models with recovered and restrained states suggest that the infected nodes/ spreaders will likely reach zero after a particular time.

### Comparison with conventional epidemic models

We tested the model under a simulated sample size of 300,000 nodes by setting ten infected nodes at the starting stage. The simulation was run for 100 days with lower transmission rates, an infection rate of 0.1, and a recovery rate of 0.035—assuming that recovery is relatively more challenging than getting infected. Table 2 shows the susceptible and infected nodes during the starting and ending time of analysis for the proposed and conventional models. It can be observed that SI and SIS models are practically not possible in the current scenario as infecting two-thirds of the population in a network community at the same time is less probable. Moreover, the SI model states that once infected nodes stay infected forever, which is practically impossible for a single disinformation content in social networks. Similarly, the probability of a person who came out from infection returning to the infected state is less, which makes the SIS model practically less possible as the model lacks Recovery or Restrained states. Since the SEDIS model describes the pandemic nature of rumors by considering all rumors and fake news as a single pandemic, it is not feasible for epidemic modeling by considering a single misinformation.

**Table 2:** Comparison of epidemic models for social networks.

Model	Day	S	I	Peak infection
SI model	0	299,990	10	299,986
	100	14	299,986	
SIR model	0	299,990	10	170,548
	100	14	13,916	
SIS model	0	299,990	10	222,222
	100	77,778	222,222	
SEDIS model	0	299,990	10	164,606
	100	77,778	164,606	
SEDPNR model	0	299,990	5(P) + 5(N)	141,874
	100	612	21,402 (P+N)	

**Figure 9:**
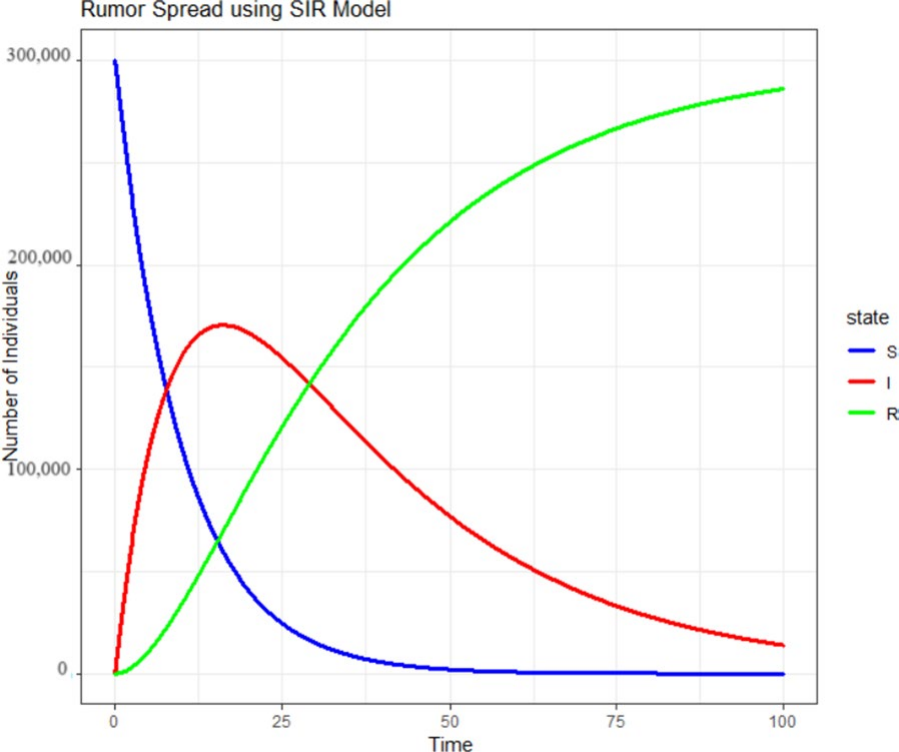
Simulation of SIR model for 300,000 individuals.

Figure 9 highlights the SIR model among 300,000 individuals where the susceptible population quickly reaches zero value. This is, however, not quickly possible in the current scenario (especially when the spreaders who start propagating the fake news have deliberate intentions to spread the content as long as he/ she needs can. Moreover, we also have to consider a common fact that a good number of people on the internet will not be active at the same time. Many of the population could miss the fake news being propagated online, even if the information is exciting and shared in their community. The significance of power law comes here as some distributions within the network do not exhibit normal behavior. Figures 3 and 4 signify the same. Based on these figures, it can be inferred that communities in countries with higher human development values experience a rapid decrease in infection rates compared to countries with lower human development index values. It should be noted, however, that this observation may only hold for countries with a significant internet penetration rate. Further research is needed to confirm this finding by including data from additional countries in the analysis.

According to this model, an individual exposed to a rumor can transition into either an Infected or Doubter state, which is determined by their psychological, social, and emotional intelligence levels. Additionally, an Exposed person can return to the Susceptible state by rejecting a rumor. Those still determining the accuracy and comprehensibility of information are considered to be in the Doubter state. According to Karl Albrecht’s work, social intelligence has five dimensions: awareness, presence, authenticity, clarity, and empathy^67^. Suppose the authenticity of the source or the information is in doubt. In that case, an individual is less likely to spread the same depending on his/her psychology, nature, choice, and mental well-being. Moreover, an individual’s sharing of a rumor is influenced by situational awareness and the clarity of the information received. These two dimensions are essential in our proposed model. Additionally, social influence, a necessary aspect of social intelligence—particularly awareness and empathy, is crucial to understanding misinformation spread. Echo chambers and central hubs create environments where people are exposed to limited perspectives, amplifying the influence of misinformation within that network^68^.

Moreover, many people turn to fact-checking websites and other digital platforms to confirm the legitimacy of information they get through online social networks^69^. Moreover, efforts by digital media platforms to prevent the spread of misinformation have increased significantly recently^70^. The Doubter node is significant in this context, as it incorporates aspects of social intelligence.

When it comes to how information dispersion and user belief states are represented, the SEDPNR model departs greatly from the SIR and SEIR models. The following are the main distinctions between the suggested SEDPNR model and the traditional SIR and SEIR epidemic models:

- SIR/SEIR is more concerned with biological processes, whereas SEDPNR is more concerned with user psychology and social effects on information.
- Positive (P) and Negative Infected (N) states, which are missing from SIR/SEIR, allow SEDPNR to record user attitude (belief/distrust).
- Restrained (R) states in SEDPNR represent people who could continue to accept false information but choose not to disclose it; this notion is absent from SIR/SEIR.

### Strategies for mitigating the spread of misinformation

The SEDPNR model is designed to address the behavioral outcomes of online users when a single misinformation is circulated within the online digital network. This model considers various factors, including the psychology of choice, and is specifically tailored for online users. The compartments in the SEDPNR model accurately depict the spread of infection and include all relevant categories of social media users. The model comprehensively explains how rumors spread on social media, considering key factors such as individual susceptibility, exposure, distrust, and recovery. This lends itself to more accurate predictions of how rumors will evolve and diffuse in these networks. By delving deeper into this model, future research can uncover the circumstances in which rumors thrive or die. With this valuable insight, strategies can be developed to intervene and mitigate the spread of misinformation. The SEDPNR model can help guide the development of debunking tactics by providing a sophisticated understanding of user behavior and information spread. Doubt’s function is one important realization. The model highlights the Doubter state (D) as an essential area for intervention. Policyholders can encourage the dissemination of factual counter-narratives that dispel people’s particular doubts. This might entail collaborating with reliable sources to provide material that dispels myths or giving people the resources to verify information independently.

Secondly, the model emphasizes the importance of social pressure and self-censorship (represented by the “restrained” state, R). The policymakers should push platforms to provide tools that encourage openness about the origins of information and identify potentially deceptive content. This can encourage users to share information more carefully and give them the authority to serve as gatekeepers against false information.

In our modeling approach, we consider the underlying structure of the digital network as a complex system that various graph models can represent. While our current model focuses on the dynamics of information spread, the network topology plays a crucial role in how information propagates. Different graph hypotheses can be applied to model social networks:

1. Random Graphs: As introduced by Erdős and Rényi^71^, these can model the unpredictable nature of connections in large networks.
2. Scale-Free Networks: Proposed by Barabási and Albert^72^, these better represent the power-law degree distribution often observed in social networks.
3. Small-World Networks: Watts and Strogatz^73^ introduced this model, which captures both high clustering and short average path lengths characteristic of many real-world networks.
4. Multiplex Networks: These can represent the multi-platform nature of modern social media interactions^74^.
5. Weighted Graphs: These can model the strength of connections between users^75^.

Future work could involve explicitly incorporating these network structures into our SEDPNR model to enhance its predictive power and applicability to various digital environments. Finally, the SEDPNR model can help design focused treatments. By calibrating the model using actual data, policymakers can forecast how various initiatives would affect the dynamics of a disinformation epidemic. This enables a more deliberate distribution of resources, emphasizing disproving the most significant false information and slowing its dissemination to the most vulnerable groups. Additionally, by utilizing sentiment data during times of crisis, the model can identify and track the propagation of rumors, informing effective public health and crisis communication strategies.

While the SEDPNR model offers valuable insights into misinformation dynamics, future research can explore several promising directions to enhance their application in controlling misinformation.

Incorporating Network Heterogeneity: Current models frequently assume homogenous networks, with nodes having identical connection patterns. Future studies can incorporate network heterogeneity, in which people have varied degrees of impact and link with others based on hobbies or demography. This would enable more accurate modeling of how disinformation spreads across online groups, including echo chambers and targeted efforts.

Integrating Fact-Checking and User Behavior: Current models frequently fail to account for interventions like fact-checking or user activities such as reporting disinformation. Future studies can look into ways to merge these aspects. This might include modeling the effect of timely and reliable fact-checking on rumor dissemination and how user actions to report or dispel misinformation can influence its spread.

Developing Mitigation Strategies: Building on the preceding lines, future research might explore more tailored mitigation techniques based on the unique traits and dynamics of disinformation transmission. This might entail creating treatments suited to certain forms of disinformation, network architecture, or user behavior.

While models can provide valuable insights, it’s important to remember that user awareness is a crucial factor in combating misinformation. Future research can play a significant role in developing educational campaigns and media literacy initiatives. These initiatives are crucial in equipping users with the skills to evaluate the information they encounter online critically. By exploring these potential future directions, misinformation models for social and digital networks can become even more powerful tools in the fight against misinformation. They can offer a deeper understanding of how misinformation spreads, inform the development of targeted mitigation strategies, and ultimately contribute to a more informed and healthy online environment.

## Conclusion and future scope

Misinformation and fake news are significant challenges in online digital networks, mainly through social media platforms, as it can be difficult for people to differentiate between genuine facts and misinformation. Despite some platforms implementing disputed tags to classify misinformation, instant messaging applications still pose a severe threat to the viral spread of misinformation. While several epidemic models have been proposed to study fake news and misinformation in digital networks, they often need to include human social intelligence dimensions.

To address this gap, we propose the SEDPNR model, which includes doubter and exposed states and divides the infected node based on the user sentiment. Our mathematical analysis supports the system’s validity, and our graphical curve with real-world data confirms the existence of a power law within the system, denoting the super-spreaders who deliberately spread disinformation, thereby connecting our model to a real-world approach. However, different digital online networks work differently, and the information users see can vary depending on the social network and usage patterns. The critical findings of the studies include the following; When it comes to the dissemination of false information, the SEDPNR model displays a power-law distribution that closely matches empirical data. This implies that a small number of compelling people or sources can significantly influence the scale of the epidemic. In contrast to regular times, our simulations reveal a rise in the “Negative Infected” (N) condition during a crisis. This is consistent with the fact from the actual world that unfavorable feelings and mistrust of information tend to increase during times of crisis. Furthermore, the population as a whole (S + E + D + P + N + R) is constant as the model is a bounded system. This preserves the stability of the model and captures the fact that people cannot be “restrained” from sharing knowledge or kept infected eternally. However, as with any model, the specific findings of the SEDPNR model depend on the chosen parameters and calibration data. Real-world misinformation campaigns can be complex, and the model’s accuracy can benefit further refinement. Social networks like Facebook present content based on user’s interests and the postings they spend the most time on, whereas platforms like Twitter and WhatsApp work on entirely different phenomena. Because this variance might alter the exposed, doubter, and infection rates in different networks, it is critical to understand the stiffness of each global community when analyzing rumor dissemination.

The model’s transition between user belief states (like Doubter to Infected) is a complex process, and the model’s current approach could benefit from more empirical backing. One of the main limitations of the model is that the model treats transitions as binary events based on encountering information or social influence. Real-world transitions are likely influenced by various factors like the information’s content, source credibility, user experience, and individual psychology. Moreover, validating the model requires data on user belief states, which can be challenging to collect ethically on social media platforms. Additionally, self-reported data might need to reflect internal beliefs accurately. By considering this, future work can be focused on conducting controlled studies where users are exposed to different types of misinformation, which can help understand how factors like content, source, and user experience influence transitions between belief states. Additionally, analyzing user engagement metrics (likes, shares, comments) alongside sentiment analysis can provide indirect insights into user belief states. In future work, we plan to implement stiffness analysis using real-world data and our formulated models. With the help of this generated model, additional research by text mining social network data and conducting extensive surveys will provide rewarding results.

## Data Availability

The data generated and/or analyzed during the current study are available in the Github repository, on the following links: $\cdot$https://github.com/sreeraag2007/BrahmapuramFire$\cdot$https://github.com/sreeraag2007/SentimentAnalysis_Results/.
